# A New Safety Concern for Glaucoma Treatment Demonstrated by Mass Spectrometry Imaging of Benzalkonium Chloride Distribution in the Eye, an Experimental Study in Rabbits

**DOI:** 10.1371/journal.pone.0050180

**Published:** 2012-11-27

**Authors:** Françoise Brignole-Baudouin, Nicolas Desbenoit, Gregory Hamm, Hong Liang, Jean-Pierre Both, Alain Brunelle, Isabelle Fournier, Vincent Guerineau, Raphael Legouffe, Jonathan Stauber, David Touboul, Maxence Wisztorski, Michel Salzet, Olivier Laprevote, Christophe Baudouin

**Affiliations:** 1 INSERM, U968, Paris, France; 2 UPMC Univ Paris 06, UMR_S 968, Institut de la Vision, Paris, France; 3 CNRS, UMR_7210, Paris, France; 4 Centre Hospitalier National d’Ophtalmologie des Quinze-Vingts, INSERM-DHOS CIC 503, Paris, France; 5 Centre de recherche de Gif, Institut de Chimie des Substances Naturelles, CNRS, Gif-sur-Yvette, France; 6 Laboratoire d’Intégration des Systèmes et des Technologies, CEA-LIST, Gif-sur-Yvette, France; 7 Laboratoire de Spectrométrie de Masse Biologique, Fondamentale et Appliquée, EA 4550, Université Lille Nord de France – Université Lille 1, Villeneuve d’Ascq, France; 8 Imabiotech Campus Cité Scientifique, Villeneuve d’Ascq, France; 9 Chimie Toxicologie Analytique et Cellulaire, EA 4463, Faculté des Sciences Pharmaceutiques et Biologiques, Université Paris Descartes, Paris, France; 10 Université Versailles Saint-Quentin-en-Yvelines, Versailles, France; 11 Assistance Publique - Hôpitaux de Paris Hôpital Ambroise Paré, Service d’Ophtalmologie, Boulogne-Billancourt, France; Univeristy of Melbourne, Australia

## Abstract

We investigated in a rabbit model, the eye distribution of topically instilled benzalkonium_(BAK) chloride a commonly used preservative in eye drops using mass spectrometry imaging. Three groups of three New Zealand rabbits each were used: a control one without instillation, one receiving 0.01%BAK twice a day for 5 months and one with 0.2%BAK one drop a day for 1 month. After sacrifice, eyes were embedded and frozen in tragacanth gum. Serial cryosections were alternately deposited on glass slides for histological (hematoxylin-eosin staining) and immunohistological controls (CD45, RLA-DR and vimentin for inflammatory cell infiltration as well as vimentin for Müller glial cell activation) and ITO or stainless steel plates for MSI experiments using Matrix-assisted laser desorption ionization time-of-flight. The MSI results were confirmed by a round-robin study on several adjacent sections conducted in two different laboratories using different sample preparation methods, mass spectrometers and data analysis softwares. BAK was shown to penetrate healthy eyes even after a short duration and was not only detected on the ocular surface structures, but also in deeper tissues, especially in sensitive areas involved in glaucoma pathophysiology, such as the trabecular meshwork and the optic nerve areas, as confirmed by images with histological stainings. CD45-, RLA-DR- and vimentin-positive cells increased in treated eyes. Vimentin was found only in the inner layer of retina in normal eyes and increased in all retinal layers in treated eyes, confirming an activation response to a cell stress. This ocular toxicological study confirms the presence of BAK preservative in ocular surface structures as well as in deeper structures involved in glaucoma disease. The inflammatory cell infiltration and Müller glial cell activation confirmed the deleterious effect of BAK. Although these results were obtained in animals, they highlight the importance of the safety-first principle for the treatment of glaucoma patients.

## Introduction

Glaucoma is a severe optic neuropathy leading to blindness without treatment and affecting more than 70 million people worldwide. This insidious disease is the main cause of irreversible blindness and is associated with increased intraocular pressure due to a resistance in the trabecular meshwork outflow pathway of aqueous humor [1]. Once diagnosed, treatment must be taken throughout life to prevent or halt retinal ganglion cell loss and visual deterioration. Consequently, patients have to be treated for the rest of their life with intraocular pressure (IOP)-lowering multi-dose eye drops (1). Most of these eye drops contain a preservative: the most commonly used is benzalkonium chloride (BAK), a quaternary ammonium salt composed of a mixture of benzododecinium C_21_H_38_N^+^ (BAK C_12_) and myristalkonium C_23_H_42_N^+^ (BAK C_14_) chlorides. BAK is a cationic surfactant and tensioactive compound, acting as a detergent for the lipid layer of the tear film as well as for the lipids of cell plasma membranes. It is reputed to increase bioavailability or penetration of active compounds and can be used as a penetration enhancer [2], [3]. At a concentration ranging from 0.004 to 0.2% in eye drops, this preservative is required by pharmacopeia guidelines to prevent the multidose eye drop containers from bacterial and fungi contamination [4], [5]. Although it has the advantage of inducing fewer allergic-type side effects and of being relatively well tolerated, it has been reported to induce ocular surface disorders combining irritation, inflammation and cell death processes, especially in long-term treatment [6]. There is a growing body of evidence that BAK induces apoptosis, oxidative stress and inflammation on the ocular surface epithelia, unlike antiglaucoma active compounds that have been demonstrated to be safe for epithelial cells [6]. While the deleterious effects of BAK could be negligible for a short-term treatment, they need to be considered for long-term or repeated treatment such as in chronic open-angle glaucoma. In this case, patients are most often treated for the rest of their life, often with several BAK-containing eye drops, since about 40% of patients require multiple therapies to control their IOP and prevent further optic nerve damage [7]. The ocular surface structures, tear film, cornea, conjunctiva as well as the eyelids are the tissues most apparently involved in treatment tolerance. BAK-containing antiglaucoma eye drops have been reported to cause disruption of the blood–aqueous barrier inducing cystoid macular edema following cataract surgery [8].

**Figure 1:**
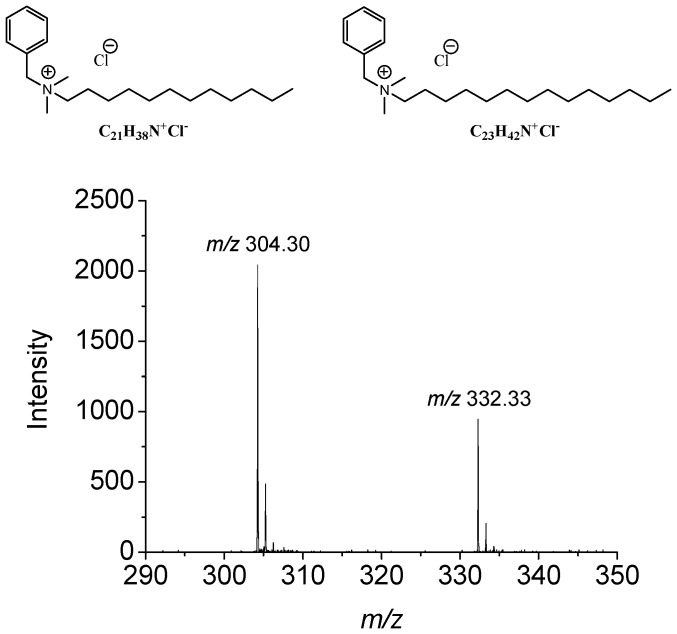
Chemical structures and MALDI-TOF spectra. Chemical structure and MALDI-TOF spectrum of benzalkonium homologs used in this study, BAK C_12_ and BAK C_14_, are presented with their respective MALDI-TOF spectrum in positive ion mode. The BAK solution instilled in the rabbit eyes contained two thirds of BAK C_12_ (*m/z* 304.30) and one third of BAK C_14_ (*m/z* 332.33).

Little is known about BAK penetration and distribution in the eye. A study reported its presence in the conjunctiva after a single drop of BAK up to 7 days after instillation [9]. In an in vivo study, Chou et al. found abnormal electroretinograms (ERG) with a reduction in the a- and b-wave amplitudes in rabbits receiving subconjunctival injections of beta-blockers associated with BAK, showing a pathway for BAK to reach the eye’s posterior segment [10]. Recently, Garrett and colleagues found BAK in the outer periphery of a human donor eye using a mass spectrometry imaging (MSI) technique [11]. In fact, the use of MSI to study tissue distribution is continuously growing. In contrast to the autoradiography technique classically used for tissue distribution analysis of a radio-labeled compound, MSI by matrix-assisted laser desorption/ionization (MALDI) is a powerful label-free technique that can identify a compound as well as its metabolites by detecting specific peaks in their mass spectra with a histologic resolution of about 50 µm [12], [13], [14], [15]. It has already been used for pharmacokinetics studies of drug distribution in the eye [16]. Actually, in the particular case of glaucoma treatment, it is crucial to ensure the absence of adverse effects, not only for the ocular surface tissues to promote tolerance and compliance, but also for deeper ocular tissues such as the trabecular meshwork, lens, retina or optic nerve to preserve visual function. Our objective was therefore to investigate BAK penetration using MSI in a rabbit model. This enabled us to see whether was able to reach sensitive ocular areas involved in glaucoma physiopathology that could compromise treatment efficacy and threaten visual function over the long term.

**Figure 2:**
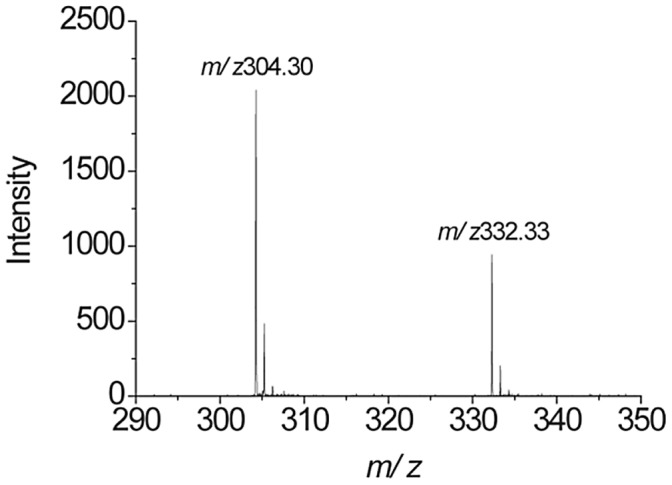
MALDI-TOF imaging of whole eye section of a control rabbit. MALDI-TOF imaging shows the absence of benzalkonium chloride (BAK) in the control eye. (a) Histology image of an adjacent cryosection stained with hematoxylin-eosin (HE) showing three areas of interest: cornea (area 1), nasal iridocorneal angle (area 2) and near to the optic nerve (area 3). (b, c) Overlays between HE and MALDI-TOF images of BAK C_12_ and C_14_ eye distributions at *m/z* 304.32 and 332.36, respectively. Intensities of the ions are represented in colour, based on the intensity scale provided (from black to white). Field of view 18× 6 mm. (d, e, f) MALDI-TOF mass spectra extracted from areas 1, 2 and 3, respectively confirming the absence of BAK C_12_ and C_14_.

## Materials and Methods

### Chemicals

HPLC-grade acetonitrile and water were purchased from BioSolve (Valkenswaard, Netherlands) or Sigma-Aldrich (Saint-Quentin Fallavier, France). Alpha-cyano-4-hydroxycinnamic acid (CHCA), trifluoroacetic acid (TFA), hematoxylin, eosin, ethanol and benzalkonium chlorides (BAK) were purchased from Sigma-Aldrich (Saint-Quentin Fallavier, France). The chemical structures of benzalkonium homologs BAK C_12_ and BAK C_14_ (2∶1) used in this study are presented in figure 1.

### Animals

All rabbits used in this study (n = 3 in each group) were white New Zealand albino rabbits weighing 2–2.5 kg and were purchased from Cegav, St Mars-d’Egrenne, France. They were housed individually in stainless-steel wire-bottom cages in environmentally controlled rooms with a 12-h light/12-h dark cycle, a 30–70% humidity range and a22–24°C temperature range. They were allowed to drink tap water and to eat *ad libitum*.

### Ethics Statement

This study was conducted in accordance with the Association for Research in Vision and Ophthalmology (ARVO) statement for the Use of Animals in Ophthalmic and Vision research and was approved by the local ethics committee for animal experimentation at the Faculty of Pharmaceutical and Biological Sciences, Sorbonne Paris Cité University.

**Figure 3:**
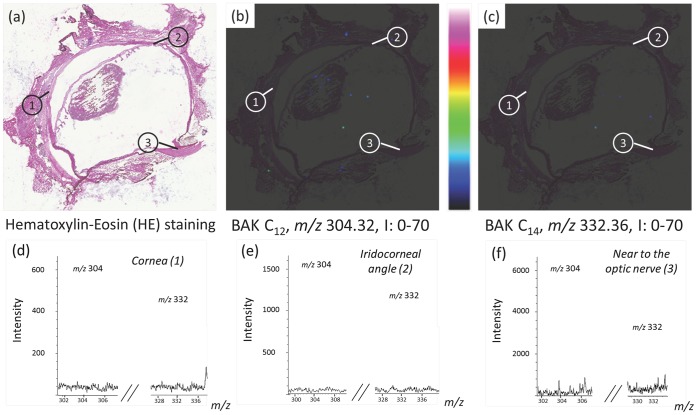
MALDI-TOF imaging of whole eye section of a rabbit instilled twice a day with one drop of 0.01% benzalkonium chloride (BAK) for 5 months. MALDI-TOF imaging shows the BAK distribution in a BAK-treated eye. (a) Histology image of an adjacent cryosection stained with hematoxylin-eosin (HE) showing three areas of interest: cornea (area 1), nasal iridocorneal angle (area 2) and optic nerve area (area 3). (b, c) Overlays between HE staining and MALDI-TOF ion images of BAK C_12_ and C_14_ distributions in whole eye section at *m/z* 304.30 and 332.33, respectively. (d, e) MALDI-TOF ion images of BAK C_12_ and C_14_ distributions at *m/z* 304.30 and 332.33, respectively. Intensities of the ions are represented in colour, based on the intensity scale provided (from black to white). Field of view 16×15 mm. (f, g, h) MALDI-TOF mass spectra extracted from areas 1, 2 and 3, respectively, showing BAK C_12_ and C_14_ ion peaks.

### Study Design

New Zealand albino rabbits were instilled with BAK solutions in PBS containing 65.7% C_12_ and 30.7% C_14_ homologs; a MALDI mass spectrum is shown in Figure 1. Two different toxicity models were used in this study. The first one, called the Low Chronic model (LCm) was intended to simulate chronic use with a low BAK concentration for a long time, *i.e.*, instillation of one drop of 0.01% BAK concentration, the most commonly used concentration in eye drops, twice a day for 5 months. The second model, called the High Sub-Chronic model (HSCm) was used to mimic a subchronic use at a high concentration for a shorter time with a 0.2% BAK concentration once daily for 1 month. This latter model was selected in order to maximize the toxic effect and to be able to see the eye penetration pathways. Non-instilled rabbits were used as controls.

**Figure 4:**
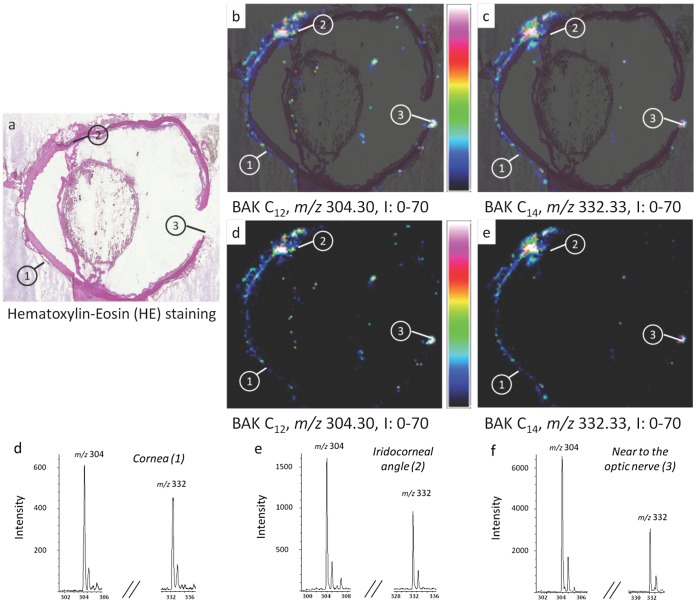
Round-robin experiment using the AutoFlex speed LRF MALDI-TOF mass spectrometer (ImaBiotech). MALDI-TOF imaging generated by the AutoFlex speed LRF MALDI-TOF mass spectrometer (ImaBiotech) shows the BAK distribution in whole eye section of a rabbit instilled once a day with one drop of 0.2% benzalkonium chloride (BAK) for 1 month. (a) Histology image of an adjacent cryosection stained with hematoxylin-eosin (HE) showing three areas of interest: cornea (area 1), nasal iridocorneal angle (area 2) and optic nerve area (area 3). (b, c) Overlays between HE staining and MALDI-TOF ion images of BAK C_12_ and C_14_ distributions in whole eye section at *m/z* 304.30 and 332.33, respectively. (d, e) MALDI-TOF ion images of BAK C_12_ and C_14_ distributions in whole eye section at *m/z* 304.30 and *m/z* 332.33, respectively. Intensities of the ions are represented in colour, based on the intensity scale provided (from black to white). Field of view 8×10 mm. (f, g, h) MALDI-TOF mass spectra extracted from areas 1, 2 and 3, respectively, showing BAK C_12_ and C_14_ ion peaks.

### Sample Processing

After sacrifice, the eyes were quickly enucleated and embedded in tragacanth gum (Alfa Aesar, Schiltigheim, France) and frozen at −80°C. Serial cryosections (14 µm thick) were cut at −30°C with a CM3050-S cryostat (Leica Microsystems SA, Nanterre, France) and deposited on stainless steel plates for MALDI MSI experiments. Before MSI analyses, the samples were dried in a vacuum, at a pressure of a few hectopascals for 15 min, with no further treatment. Optical images were recorded with an Olympus BX51 microscope (Olympus, Rungis, France) equipped with ×1.25 to ×50 lenses and a Color View I camera, monitored by Cell^B^ software (Soft Imaging System, GmbH, Münster, Germany). Cryosections of rabbit eyes adjacent to sections dedicated to MSI were stained with hematoxylin-eosin (HE) and analyzed using a DM5000 optic microscope (Leica Microsystems SA, Nanterre, France) in order to align the MS images with histology patterns.

**Figure 5:**
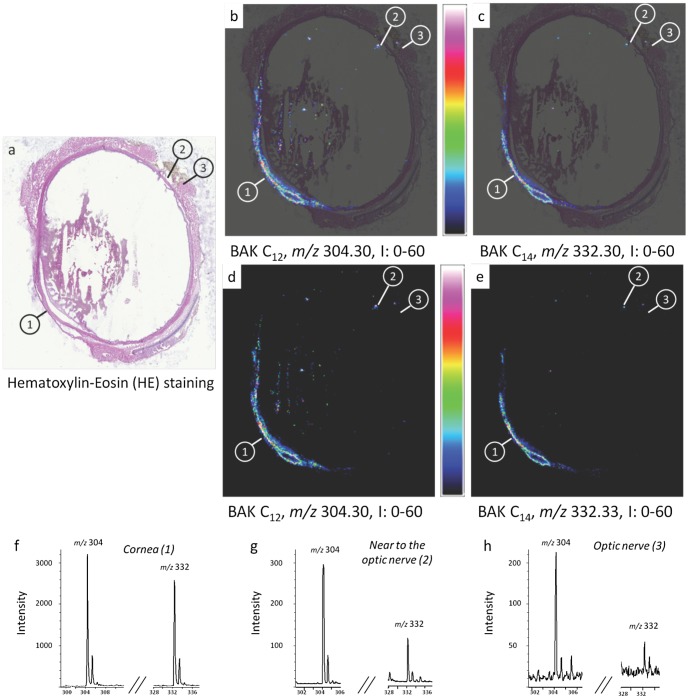
Round-robin experiment using the 4800 MALDI-TOF/TOF mass spectrometer (ICSN-CNRS). MALDI-TOF imaging generated by the 4800 MALDI-TOF/TOF mass spectrometer (ICSN-CNRS) shows the BAK distribution in whole eye section of a rabbit instilled once a day with one drop of 0.2% benzalkonium chloride (BAK) for 1 month. (a) Histology image of an adjacent cryosection stained with hematoxylin-eosin (HE) showing three areas of interest: cornea (area 1), near to optic nerve area (area 2) and optic nerve (area 3). (b, c) Overlays between HE staining and MALDI-TOF ion images of BAK C_12_ and C_14_ distributions in whole eye section at *m/z* 304.32 and *m/z* 332.36, respectively. (d, e) MALDI-TOF ion images of BAK C_12_ and C_14_ distributions in whole eye section at *m/z* 304.30 and *m/z* 332.33, respectively, with intensity scale from 0 to 714. Intensities of the ions are represented in color, based on the intensity scale provided (from black to red). Field of view 18×23 mm. (f, g, h) MALDI-TOF mass spectra extracted from areas 1, 2 and 3, respectively, showing BAK C_12_ and C_14_ ion peaks.

### MALDI Mass Mpectrometry Imaging

MALDI mass spectrometry images were acquired in two different laboratories, which are named in the following text ImaBiotech and CNRS-ICSN, respectively.

#### MALDI mass spectrometry imaging (ImaBiotech)

Optical images of each stained section were acquired using an HP scan (Hewlett-Packard, Palo Alto, CA, USA). CHCA at 10 mg/mL in acetonitrile/water/trifluoroacetic acid (ACN/H_2_O/TFA, 60/40/0.1, V/V/V) was used as the matrix solution. The matrix solution was sprayed onto the eye sections using the SunCollect automatic sprayer (SunChrom, Friedrichsdorf, Germany). Each layer was sprayed at a 20-µL/min flow rate, and 15 layers were overlaid to achieve a homogeneous matrix coating. MS images were acquired with an AutoFlex speed LRF MALDI-TOF mass spectrometer (Bruker Daltonics, Bremen, Germany) equipped with a Smart beam II laser used at a repetition rate of 1000 Hz. All instrumental parameters were optimized before the imaging experiment on standard samples of BAK C_12_ and C_14_ at *m/z* 304.30 and *m/z* 332.33, respectively. Positive ion mass spectra were acquired within the 100- to 1000-*m/z* range. The mass spectrometer was operated in the reflectron mode and the mass spectrum obtained for each image position corresponds to the averaged mass spectra of 700 consecutive laser shots at the same location. Two image raster steps were selected: (1) 150 µm for MS imaging of eye sections from the control rabbit (Figure 2) and from a rabbit treated for 5 months with 0.01% BAK solution (Figures 3 and 4) and (2) 80 µm for the rabbit treated 1 month with 0.2% BAK solution (Figure 5). Flex Control 3.0 and Flex Imaging 2.1 software packages (Bruker Daltonics, Bremen, Germany) were used to control the mass spectrometer, set imaging parameters and visualize imaging data. After MALDI image acquisition, the matrix was washed off the sections in 100% methanol and conventional hematoxylin-eosin (HE) staining was performed.

**Figure 6:**
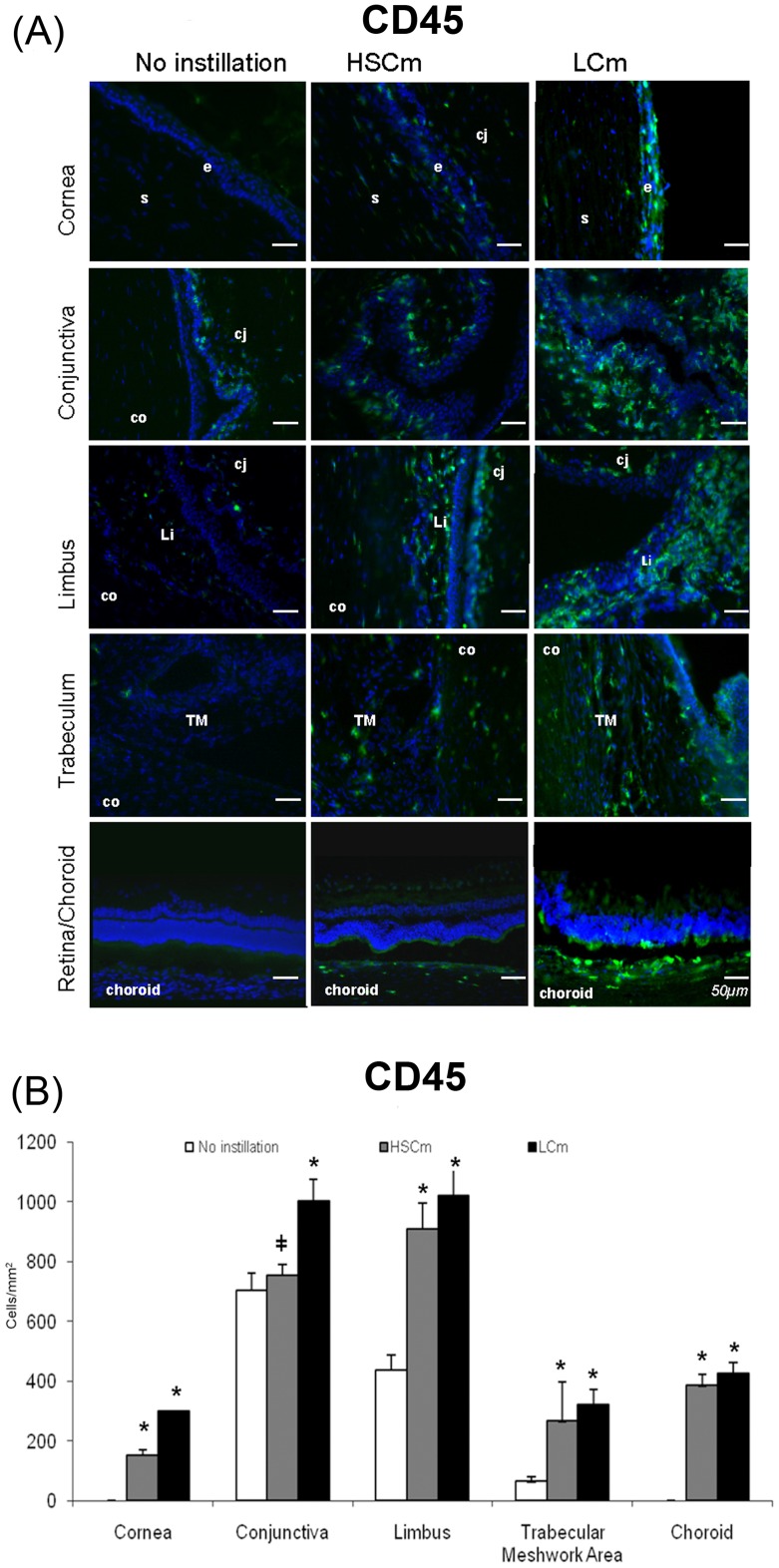
CD45-positive cell infiltration. ( A) Immunofluorescence staining of leucocytes with CD45 (in green) in rabbit eye cryosections in normal noninstilled rabbit eyes compared with rabbit eye instilled with BAK 0.01% twice a day for 5 months (Low Chronic model, LCm) and 0.2% once a day for 1 month (High Sub-Chronic model, HSCm). Nuclei are stained in blue with DAPI. Scale bar, 50 mm. Cj: conjunctiva; Co: cornea; Li: limbus; s: corneal stroma; e: superficial epithelium; TM: trabecular meshwork. (B) Histogram of CD45 positive cells count (mean cells/mm^2^±SD) *P<0.001 compared with the normal eye; I *P*<0.0001 HsCm versus LCm.

#### MALDI mass spectrometry imaging (ICSN, CNRS)

CHCA matrix solution at 10 mg/mL concentrations in acetonitrile/water/trifluoroacetic acid (ACN/H_2_O/TFA, 60/40/0.1, V/V/V) was prepared for positive ion mode analysis. Rabbit eye sections were homogeneously covered by matrix using a TM-Sprayer (HTX-Imaging, Carrboro, NC, USA). In this system, the nozzle/air spray system is heated to120°C and coupled to an isocratic pump, which provides a constant flow rate of 240 µL/min of matrix solution. The MALDI target plate is anchored on an x–y axis stage, and matrix coating is achieved by moving the sample plate under the fixed nozzle/air system at a linear velocity of 120 cm/min. Data were acquired with a 4800 MALDI TOF/TOF mass spectrometer (AB SCIEX, Les Ulis, France) equipped with a 200-Hz tripled-frequency Nd/YAG pulsed laser (355 nm) and an electrostatic mirror, providing a routine mass resolution of about 15,000 in MS mode. The data were acquired in the positive reflectron ion mode at an accelerating potential of 20 kV and a delayed extraction of450 ns (80%). Internal mass calibration was achieved using known ions present at the sample surface such as *m/z* 379.09 (protonated CHCA dimer) or 877.73 (triacylglycerol, [TG (54∶7) + H]^+^). The number of laser shots per pixel was set to 120. The distance between two adjacent pixels was set to 80 µm, which roughly corresponds to the laser spot diameter and which thus defines the lateral resolution. This leads to acquisition times of several hours, depending on the surface size analyzed. The images were recorded using 4000 Series Imaging software (www.maldi-msi.org, M. Stoeckli, Novartis Pharma, Basel, Switzerland), processed using Tissue View software (AB Sciex, Les Ulis, France), and are presented in Figure 5.

**Figure 7:**
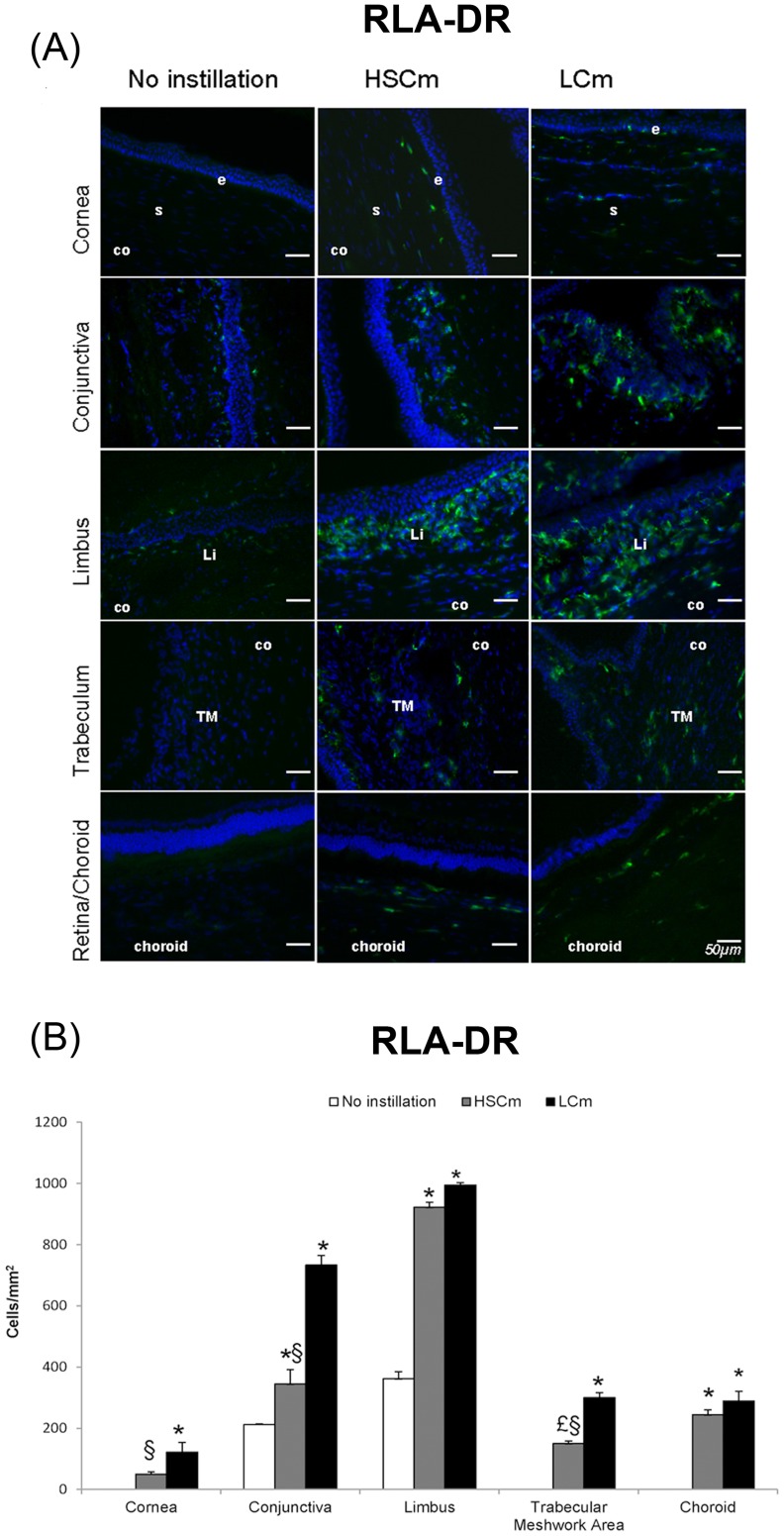
RLA-DR-positive cell infiltration. (A) Immunofluorescence staining of antigen presenting cells expressing RLA-DR (in green) in rabbit eye cryosections in normal non instilled rabbit eyes compared with rabbit eye instilled with BAK 0.01% twice a day for 5 months (Low Chronic model, LCm) and 0.2% once a day for 1 month (High SubChronic model, HSCm). Nuclei in blue are stained with DAPI. Scale bar, 50 mm. Cj: conjunctiva; Co: cornea; Li: limbus; s: corneal stroma; e: superficial epithelium; TM: trabecular meshwork. (B) Histogram of RLA-DR positive cells count (mean cells/mm^2^±SD) *P<0.001 compared with the normal eye or £ *P*<0.001 compared with the normal eye; § *P*<0.01 HsCm versus LCm.

### Immunohistological Study in Cryosections and Positive Cell Counts

Whole rabbit eye cryosections were fixed in 4% PFA for 15 mn at 4°C, washed in PBS with 1% BSA, permeabilized with 0.01%-diluted Triton X100® (Sigma Chemical Company, St Louis, MO, USA) for 5 min and incubated for 2 h at 4°C with mouse immunoglobulins directed against rabbit pan-leukocyte antigen CD45 (1∶50; CBL1412, Cymbus Biotechnology, Chandlers Ford, UK), rabbit MHC Class II Leukocyte Antigen RLA-DR (1∶100; RDR34, Beckman coulter, Miami, FL, USA) and vimentin (V9; 1/50, Dako, Glostrup, Denmark)in order to detect inflammatory cell infiltration: leukocytes, antigen-presenting cells and Müller cell activation, respectively. Normal mouse IgG (Beckman Coulter, Miami, FL, USA) were used as negative controls of fluorescence. Regarding vimentin, it is normally expressed by epithelial cells, keratocytes and fibroblasts but is highly expressed by mesenchymal infiltrating cells [17], mainly immune cells, and was used here to confirm the inflammatory infiltration and also to investigate the level of Müller glial cell activation [18]. Sections were then incubated with the secondary antibody (Alexa Fluor®488 anti-mouse immunoglobulin, 1∶500) for 1 h. After three washes in PBS, they were incubated in DAPI for 3 min (Sigma-Aldrich) to stain the nuclei. Slides were then mounted in an antifade medium (Vectashield; Vector Laboratories). Images were digitized using an epifluorescence microscope DM5000 (Leica, Wetzlar, Germany). Immunopositive cells were counted on cryosections from three rabbits using a 100×100-µm reticule in the cornea, conjunctiva, limbus, trabecular meshwork area and retina.

**Figure 8:**
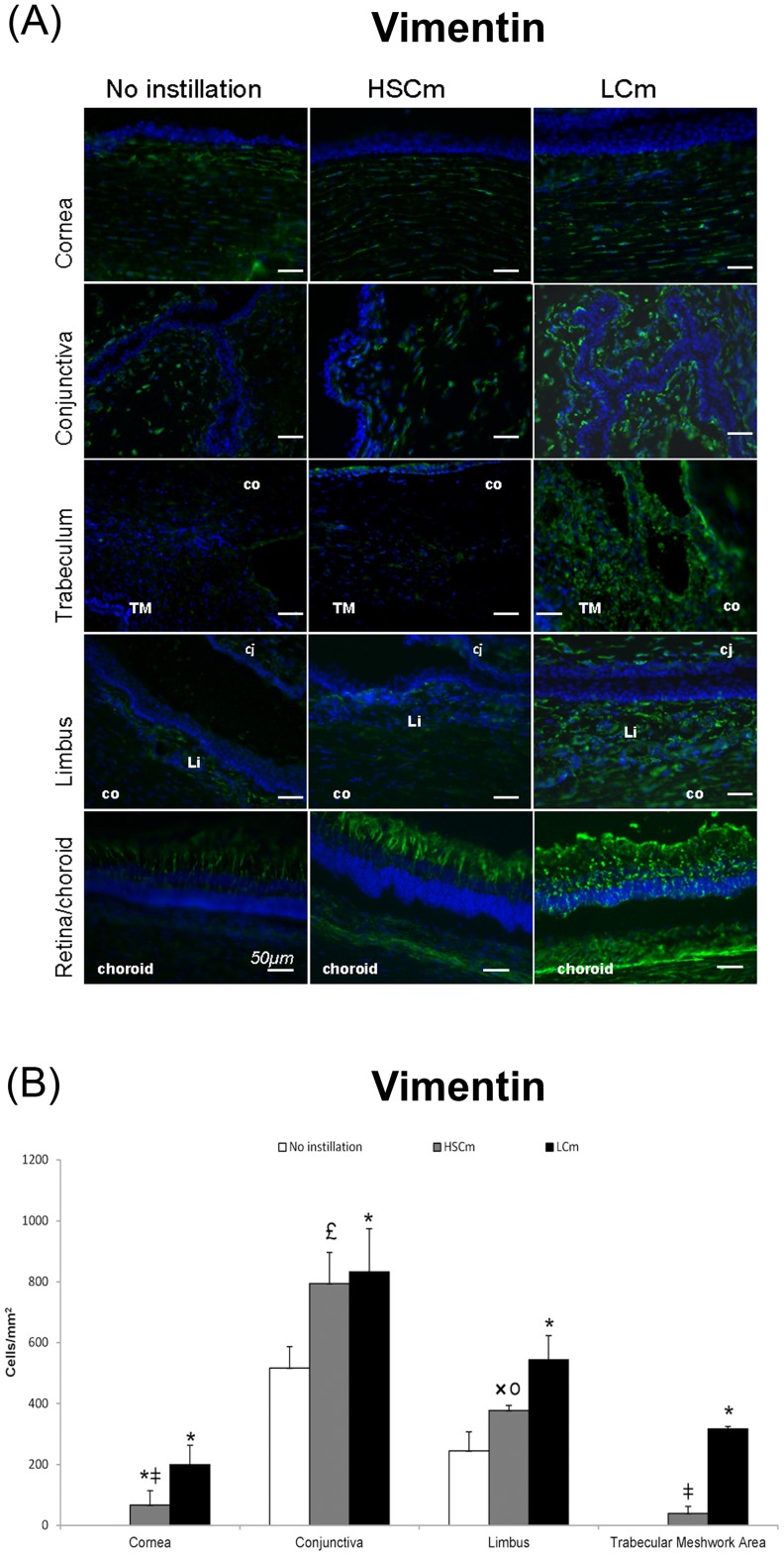
Vimentin-positive cell infiltration. (A) Immunofluorescence stainings of vimentin (in green) in rabbit eye cryosections in normal non instilled rabbit eyes compared with rabbit eye instilled with BAK 0.01% twice a day for 5 months (Low Chronic model, LCm) and 0.2% once a day for 1 month (High SubChronic model, HSCm). Nuclei in blue are stained with DAPI. Scale bar, 50 mm. Cj: conjunctiva; Co: cornea; Li: limbus; s: corneal stroma; e: superficial epithelium; TM: trabecular meshwork. (B) Histogram of vimentin positive cells count (mean cells/mm^2^±SD) *P<0.001 compared with the normal eye or £ *P*<0.001 or × *P*<0.005 compared with the normal eye; I *P*<0.0001 or ○ *P*<0.0001 HsCm versus LCm.

### Statistical Analysis

The results are expressed as means ± standard errors (SE). The groups for analysis were compared using factorial analysis of variance (ANOVA) followed by the Fisher method (GraphPad Software, La Jolla, CA, USA).

## Results

### Identification of BAK C_12_/C_14_ Homolog Mass Spectra

A MALDI mass spectrum of BAK solution was first recorded as a reference sample. Two intense ion signals at *m/z* 304.30 (BAK C_12_) and *m/z* 332.32 (BAK C_14_) were detected with a relative ratio of 2 to 1 corresponding to the relative concentration in the solution (Figure 1). Then sections of non-instilled normal rabbit eyes were analyzed according to the two MSI protocols (Figure 2). No signal corresponding to BAK C_12_ or C_14_was detected in the ion images or in the extracted mass spectra of the cornea (area 1), nasal iridocorneal angle (area 2) and near the optic nerve (area 3). This experiment clearly indicated that the control sample was not contaminated by BAK and that no natural product in the eye could interfere with BAK signals. For all following MSI experiments, we used complete MS images with corresponding adjacent HE-stained histology images (a in all figures) and we constructed overlays (b, c in all figures) of HE staining and BAK ion images (d, e in all figures) in order to visualize the anatomical structures of the eye. We particularly focused on three areas of interest: (1) the ocular surface with cornea and conjunctiva as the first ocular defense, (2) the iridocorneal angle and (3) the optic nerve area, the two ocular structures involved in glaucoma. Parts of the mass spectra are presented showing the peaks of BAK C_12_ and C_14_.

In instilled rabbit eyes–with one drop twice a day for 5 months of 0.01% BAK (LCm; Figure 3) or with one drop a day for 1 month of 0.2% BAK (HsCm; Figure 4) – BAK was detected in the cornea, the conjunctiva and the limbus, as expected with a topically applied compound. Furthermore, concerning the Low Chronic Model (LCm), similar results have been obtained between the 4800 MALDI-TOF/TOF and the AutoFlex speed LRF MALDI-TOF mass spectrometers. Interestingly, the presence of BAK was also found in deeper structures such as the sclera, near the trabecular meshwork, the filter primarily impaired in glaucoma and increasing intraocular pressure, and finally the optic nerve and its surrounding area, the structure that antiglaucoma treatments specifically aim at protecting. Some hot spots were also observed in the lens showing BAK penetration *via* a transcorneal/aqueous humor pathway (Figure 4). Hot colored spots were also seen surrounding the eyeball, this peripheral localization suggesting that the conjunctival/scleral route could be another BAK penetration pathway to reach the retina. Moreover, a series of images were acquired from adjacent sections of an animal to assess the repeatability of the results. Supplementary figures of the MS images of whole eyes are given to show such consistency (Figures S1 A, B). More than a dozen of full eye images were performed on the three groups that received the different treatments. Other images were taken by focusing only on very specific areas of the eye (cornea, iridocorneal angle, retina, optic nerve region) and were repeatedly verified, in order to assess the consistency of the results.

### Round-Robin Experiments

A round-robin study on two adjacent sections was set up by two laboratories involved in the study (ImaBiotech and ICSN, CNRS) using different sample preparation methods, mass spectrometers and data analysis software, as described above, in order to test the robustness of the entire MSI method independently of the instrumentation. A set of four adjacent eye sections was prepared. The MALDI matrix was deposited by the Sun Collect sprayer for two sections and by the TM-Sprayer for two additional sections. Then two sections prepared using each method were analyzed by each spectrometer, *i.e.*, the 4800 MALDI TOF/TOF and the AutoFlex, respectively. Finally, the dedicated software was subsequently used for data analysis. Two of the images are shown in Figures 4 and 5. The results were very similar: BAK localizations were the same with the two methods, which demonstrated the robustness of MSI. These results were also validated for both toxicological models differing in the time and the concentrations used and showed similar results (Figures 2–5). Intensities in the spectra could not be compared between the two round-robin experiments (Figures 4 and 5). The intensities could nevertheless be directly compared between different areas of the same ion image. These comparisons could be made only if we could assume that the environment had no effect on BAK desorption/ionization, indicating that the same amount of BAK in different areas of the eye induced the same intensities in the spectra. Figure 4 shows that the intensity of BAK was higher in the optic nerve than in the iridocorneal angle and in the cornea, for the rabbit eye instilled twice a day with one drop of 0.01% BAK for 5 months (a factor of ∼10). An opposite variation was observed for the rabbit eye instilled once a day with one drop of 0.2% BAK for 1 month, in which BAK was more concentrated in the cornea than in the optic nerve (greater than tenfold concentration; Figures 4 and 5). Small relative variations between intensities of BAK C_12_ and BAK C_14_, compared to the spectrum shown in Figure 1, were observed, especially in the cornea (Figures 3f and 4f), possibly indicating a different penetration of BAK in cornea depending on the length of the chain.

### Immunohistological Study in Cryosections and Positive Cell Counts

In normal non instilled eyes, CD45, RLA-DR and vimentin positive cells were not found in the cornea, and the retina/choroid, only few CD45 positive cells could be observed in the trabecular meshwork area (Figures 6, 7, 8). Conversely, positive cells are normally found in the conjunctiva and the limbus.

In the eyes instilled with BAK, immunohistological examinations clearly showed globally higher expressions of the markers tested in the LCm than in the HsCm, except for the retina/choroid area, suggesting that time had a greater effect than the BAK concentration (Figures 6, 7, 8). As RLA-DR-positive cells represent a cell subpopulation of the whole CD45-positive cell population, CD45- and RLA-DR-positive cell populations increased in the same manner but with a lower density for the RLA-DR population (Figures 6 and 7).

No positive cells could be found in the normal cornea, while CD45 and RLA-DR increased in the HsCmodel (*p*<0.001 versus control for CD45, non statistically significant increase for RLA-DR) and many positive cells were observed in the LCm (*p*<0.001 versus control; Figures 6B and 7B).

In the normal conjunctiva, CD45-positive cells were observed as expected, the conjunctiva containing lymphocytes as well as dendritic cells. These CD45-positive cells, however, increased in the two toxicological models with a tremendous increase in the LCm (*p*<0.0001 versus control and HsCm) but the difference between HsCm and control was not statistically significant (Figure 6B). The number of RLA-DR positive cells increased in the two models (*p*<0.0001 versus control) with greater values in LCm than in HsCm (*p*<0.01, figure 7B).

In the limbus, we observed an important increase in CD45 and RLA-DR positive cells (*p*<0.0001 versus control) without any difference between the two models. The same patterns were found for CD45 in the trabecular meshwork area with the same statistically significant difference with the normal rabbit control (*p*<0.0001). The number of RLA-DR positive cells increased in this area in the two models, with higher values in LCm (*p*<0.001 between HsCm and control and *p*<0.01 between LCm and HsCm).

In addition, the number of CD45 and RLA-DR positive cells was increased in the inner part of the choroid and the retinal pigment epithelium. The numeration of the cells in these areas did not differ between the two models but confirmed an increase in the two models when compared with the control (*p*<0.001).

In the corneal stroma, some vimentin positive cells were observed in the HSCm and many cells were found in the LCm (*p*<0.0001 versus control for LCm). This increase was higher for LCm than for HSCm, (*p*>0.0001) as shown on the graph (Figure 8B). We observed an increase in the number of cells expressing high levels of vimentin in the conjunctiva (Figure 8; *p*<0.0001 versus control for LCm and *p*<0.001 for HsCm with no difference between them). Numerous cells expressing high levels of vimentin were observed in the trabecular meshwork in LCm and only few positive cells in the HSCm (without difference when compared to control; *p*<0.0001 between the two models). In the limbus, positive cells increased gradually from the HSCm to the LCm. In the retina, anti-vimentin labeling extended within Müller cells from the ganglion cell layer to the outer limiting membrane with expression increasing in a time-dependent manner, reflecting an activation state of the Müller glial cells.

## Discussion

As it is relatively difficult to obtain fresh human eyes from glaucoma donors treated with BAK-containing eye drops, we undertook a toxicological study using two different conditions in New Zealand rabbits, classically considered animals of choice for toxicological studies [19]. The first model was based on the instillation of the 0.01% BAK solution, which is the concentration commonly used in eye drops, twice a day for 5 months and the other model was based on the instillation of one drop of 0.2% BAK solution once daily for 1 month. We chose this toxicological approach in order to simulate the long-term antiglaucoma treatment as much as possible according to Haber’s rule suggesting that the exposure concentration (c) multiplied by the exposure duration (t) corresponds to a constant (k) in terms of biological effect [20]. By combining the advantages of mass spectrometry (MS) and microscopy in a single experiment, the emerging MSI technology offers an extraordinarily powerful tool to understand the molecular complexity of any tissue [21]. Among the various techniques aiming to map the surface of the sample, MSI is the only analytical method capable of providing, in a single run, the spatial distribution of a wide range of molecules over the surface of a biological sample, in the present study the surface of non-fixed rabbit eye cryosections. Compared to traditional biochemical techniques based on antibodies, which are often limited by the specificity of the applied labels and the number of compounds that can be studied simultaneously, or on radiolabeling, which is difficult to carry out on living animals, MSI retrieves the molecular content of samples without requiring *a priori* knowledge of the target compounds [22]. Therefore, to characterize the spatial distribution of BAK, we used the matrix-assisted laser desorption/ionization MSI technique coupled to time-of-flight mass spectrometry (MALDI-TOF MS). Even if this method requires the deposition of an organic compound, called matrix, on top of the eye section to improve ionization, it is now well established that this ionization source offers excellent sensitivity, which is needed when examining the distribution of exogenous compounds in a tissue section. The robustness of this technique was also demonstrated by a round robin organized between two laboratories involved in the study using similar preparations but with different instrumentations. They resulted in similar observations regarding BAK detection in particular histological structures, such as the iridocorneal angle or the optic nerve area. Eventually, we evaluated the repeatability of MS imaging on more than thirty MS images from several animals, carried out in two separate laboratories, all leading to the same conclusion about the distribution of BAK and its accumulation in some histological areas of the rabbit eyes after instillation. Beyond inter-laboratory reproducibility, these results nicely demonstrate the consistency of the measurements. BAK was thus detected in ocular surface structures, cornea and conjunctiva, but also in the trabecular meshwork and optic nerve areas. Interestingly, we demonstrated that even relatively short exposure in a healthy eye might not prevent BAK from deeply entering the eye. Immunohistochemistry was indeed consistent with MSI findings, as we repeatedly found retinal changes in BAK treated eyes. This is in favor of the presence of BAK molecules that could stimulate inflammatory cell infiltration. Indeed, in the areas where BAK was observed, we showed the presence of CD45- and RLA-DR-positive cells as well as vimentin-positive cells. CD45, a pan-leukocyte marker, was used to detect any leukocyte infiltrating the tissue. HLA-DR is a MHC class II antigen expressed on antigen-presenting cells, monocytes/macrophages, B-lymphocytes and dendritic cells, and was used to confirm the presence of immune cells. Moreover, the intermediate filament vimentin, which is normally expressed in retina at the end feet of Müller cell processes, was found expressed throughout the Müller cells and up to the outer limiting membrane, as is classically observed in altered retina [18].

The immunological analyses found a greater toxic effect in the 5-month application model than in the 1-month model, suggesting that the duration of the application time has a greater impact than the BAK concentration used. This is an important finding to consider in appreciating glaucoma treatments. However, clinical studies in glaucoma patients using conjunctival impression cytology have shown that the inflammatory markers increased with the number of therapies used. Cornea and conjunctiva/sclera are the main drug absorption pathways. Drug physiochemical properties, lipophilicity, molecular size, and ionic state greatly influence absorption [23]. In addition, penetration of BAK may be enhanced in patients treated with multiple therapies and in those with an impaired ocular surface, mainly as a consequence of BAK toxicity to surface epithelia. As BAK can be detected in deep eye tissues, especially in the vicinity of the trabecular meshwork, it could be hypothesized that preserved topical antiglaucoma drugs can induce adverse effects: it may at the same time decrease IOP but cause further toxicity to this structure, resulting in an increase of aqueous outflow resistance and therefore increasing IOP. In a very recent paper, related to a clinical trial, BAK was shown to induce anterior chamber inflammation in previously untreated patients with ocular hypertension using a laser flare/cellmeter, confirming the BAK penetration inside deep ocular structures [24]. This combination of a positive IOP-lowering effect and the deleterious effects of BAK in the long run could result in apparent progressive loss of efficiency of IOP-lowering compounds. This progressive failure of treatment efficacy actually corresponds to widely observed situations in clinical practice. Indeed, nearly 40% of patients require additional eye drops in the years after diagnosis as the initial therapy fails to control pressure [7]. Additionally, as the aim of antiglaucoma drugs is to decrease IOP and consequently to protect the optic nerve, the potential presence and most likely toxicity of BAK along the optic nerve raises major sight-threatening issues. Prostaglandin F2 alpha analogs are currently the first-line treatment for reducing IOP in glaucoma patients. In a rabbit model as well as in humans, these molecules have been reported to increase optic nerve head blood flow in an IOP-reduction-independent manner, suggesting that they may reach the retina to increase blood flow [25], [26]. Moreover, Miyake proposed the term ‘pseudophakic preservative maculopathy’ for cystoid macular edema caused by antiglaucoma eyedrops, explaining that the preservative increases synthesis of prostaglandins and other inflammatory mediators and intensifies postoperative inflammation [8]. BAK could therefore cause a side effect that would be indistinguishable from the disease outcome, as the treatment would be both protective and, to a lesser but significant extent, deleterious. Such findings could account for the glaucomatous patients whose disease progresses despite good IOP control [27], [28]. Further studies with humans, whenever possible, would confirm such findings. However, we conclude that preservative-free compounds should become the first choice therapy for patients suffering from glaucoma and any other disease requiring chronic drug administration in their eyes.

## Supporting Information

Figure S1 **Repeated ion images of whole eye section of rabbit eye.** (A) Rabbit eye instilled twice a day with one drop of 0.01% benzalkonium chloride (BAK) for 1 and 5 months (Low Chronic model, LCm): two images (separated by a dotted red line) for each model (separated by a red line) showing the BAK distribution using MALDI-TOF mass spectrometry imaging: (Lines 1 and 2) MALDI-TOF ion images of BAK C_12_ and BAK C_14_ distributions in whole eye section, respectively; (Line 3) Histology images of cryosections stained with hematoxylin-eosin and unstained contrast phase optical views for each model. (B) Rabbit eye instilled once a day with one drop of 0.2% benzalkonium chloride (BAK) for 1 month (High Sub-Chronic model, HSCm): three images (separated by a dotted red line) showing BAK distribution using MALDI-TOF mass spectrometry imaging. (Lines 1 and 2) MALDI-TOF ion images of BAK C_12_ and BAK C_14_ distributions in whole eye section, respectively; (Line 3) Histology images of cryosections stained with hematoxylin-eosin (right and left) and unstained contrast phase optical views (middle).
(TIF)
