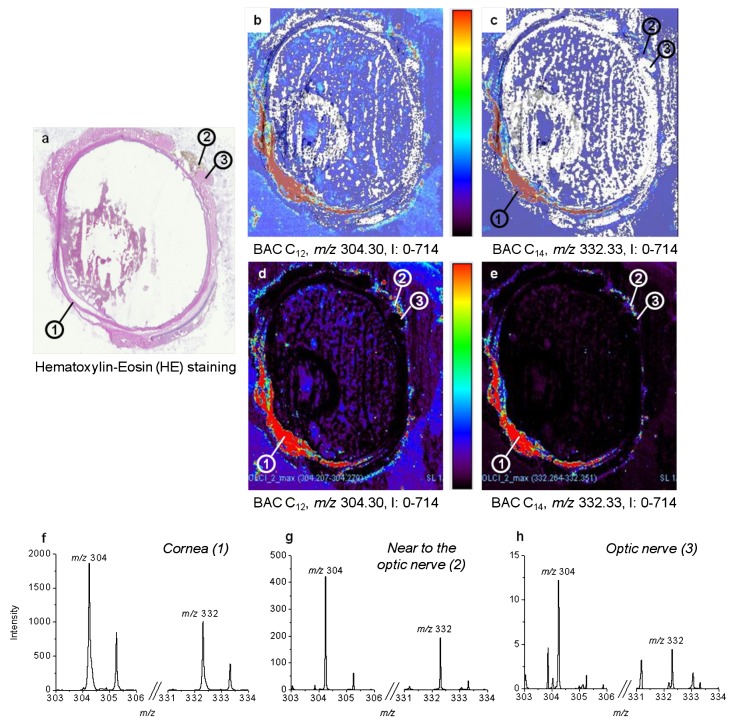# Correction: A New Safety Concern for Glaucoma Treatment Demonstrated by Mass Spectrometry Imaging of Benzalkonium Chloride Distribution in the Eye, an Experimental Study in Rabbits

**DOI:** 10.1371/annotation/b97d3c0d-b49e-40e3-a6b4-5155bd9bf3c9

**Published:** 2013-01-17

**Authors:** Françoise Brignole-Baudouin, Nicolas Desbenoit, Gregory Hamm, Hong Liang, Jean-Pierre Both, Alain Brunelle, Isabelle Fournier, Vincent Guerineau, Raphael Legouffe, Jonathan Stauber, David Touboul, Maxence Wisztorski, Michel Salzet, Olivier Laprevote, Christophe Baudouin

Due to an error introduced in the production process, the images for figures 2,3,4, and 5 were incorrectly switched. The image that appears as Figure 3 should be Figure 2, the image that appears as Figure 4 should be Figure 3, the image that appears as Figure 5 should be Figure 4, and the correct image for Figure 5 can be seen here: 

**Figure:**