# Supplementary figures and images for: A New Safety Concern for Glaucoma Treatment Demonstrated by Mass Spectrometry Imaging of Benzalkonium Chloride Distribution in the Eye, an Experimental Study in Rabbits
Source: PLoS One. 2012 Nov 27;7(11):e50180. doi: 10.1371/journal.pone.0050180 (PMC3507684; doi:10.1371/journal.pone.0050180)

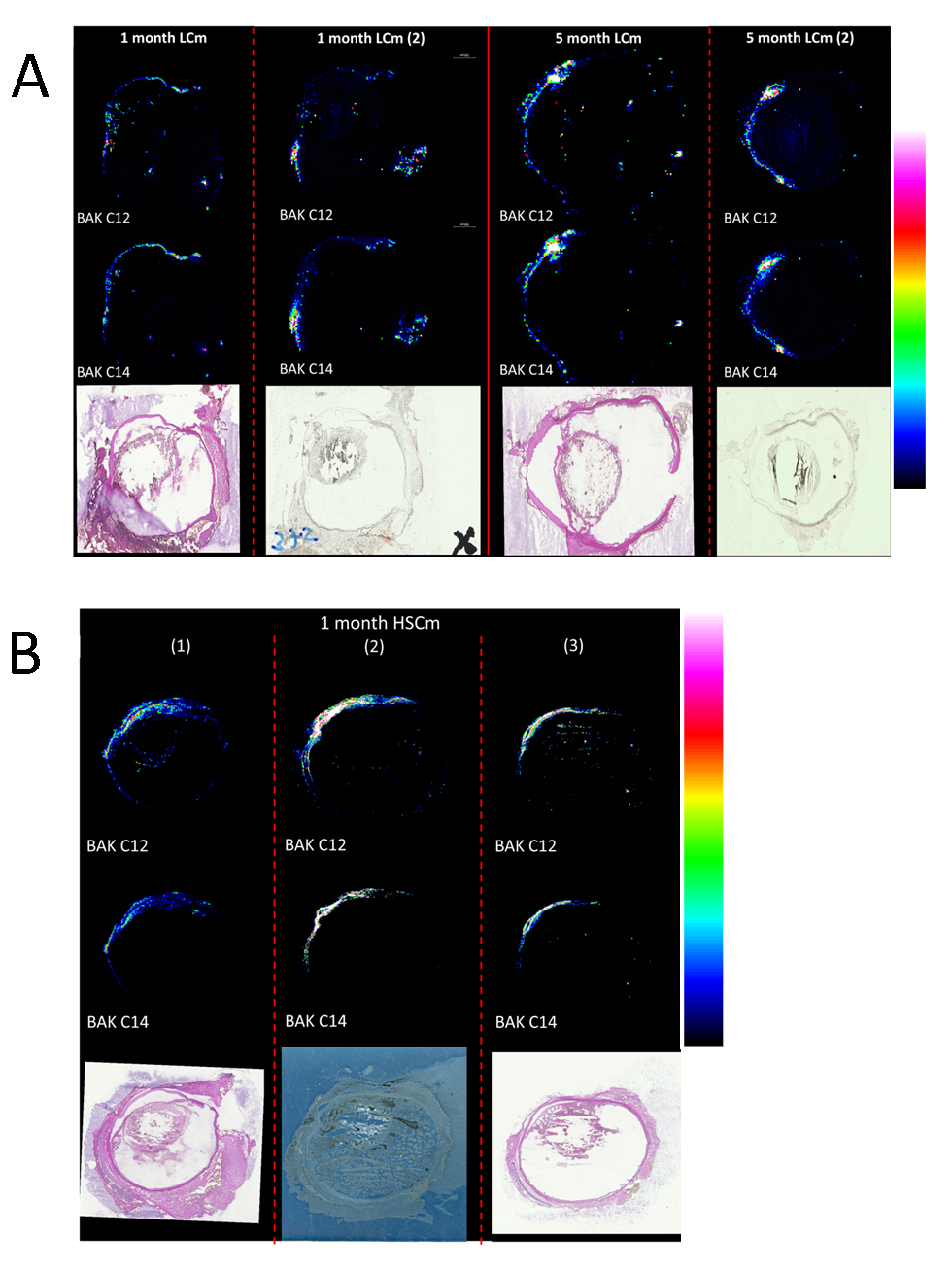

Supplement: Figure S1 — Repeated ion images of whole eye section of rabbit eye. (A) Rabbit eye instilled twice a day with one drop of 0.01% benzalkonium chloride (BAK) for 1 and 5 months (Low Chronic model, LCm): two images (separated by a dotted red line) for each model (separated by a red line) showing the BAK distribution using MALDI-TOF mass spectrometry imaging: (Lines 1 and 2) MALDI-TOF ion images of BAK C12 and BAK C14 distributions in whole eye section, respectively; (Line 3) Histology images of cryosections stained with hematoxylin-eosin and unstained contrast phase optical views for each model. (B) Rabbit eye instilled once a day with one drop of 0.2% benzalkonium chloride (BAK) for 1 month (High Sub-Chronic model, HSCm): three images (separated by a dotted red line) showing BAK distribution using MALDI-TOF mass spectrometry imaging. (Lines 1 and 2) MALDI-TOF ion images of BAK C12 and BAK C14 distributions in whole eye section, respectively; (Line 3) Histology images of cryosections stained with hematoxylin-eosin (right and left) and unstained contrast phase optical views (middle). (TIF) [file pone.0050180.s001.tif]
